# Applications of Mesenchymal Stromal Cells for Treatment of Diseases

**DOI:** 10.1002/mco2.70971

**Published:** 2026-09-08

**Authors:** Dongmei Xue, Yongsheng Li, Lei Wu

**Affiliations:** ^1^ School of Medicine Chongqing University Cancer Hospital Chongqing University Chongqing China; ^2^ Department of Medical Oncology Chongqing University Cancer Hospital Chongqing China

**Keywords:** clinical translation, immunomodulation, mesenchymal stromal cells, mitochondrial transfer, paracrine effects

## Abstract

Mesenchymal stromal cells (MSCs) are multipotent stromal cells with broad applications in regenerative medicine. The therapeutic effects of MSCs are increasingly attributed to immunomodulation, tissue repair and regeneration, paracrine effects, and mitochondrial transfer, rather than multilineage differentiation alone. Accumulating preclinical and clinical studies have demonstrated the therapeutic potential of MSCs across diverse disease settings, but clinical efficacy remains variable. However, differences in tissue source, donor age, expansion history, potency assays, and the disease microenvironment continue to limit reproducibility and clinical translation. Despite these advances, a comprehensive synthesis linking MSCs biology, mechanisms of action, disease‐specific evidence, and clinical translation is still lacking. This review summarizes the biological characteristics and heterogeneity of MSCs, and discusses the specific evidence and therapeutic mechanisms of MSCs in treating diseases such as musculoskeletal, cardiovascular, immune‐mediated, neurological, respiratory, and other diseases. We also summarize the major clinical transformation challenges of MSCs in treating various diseases. Additionally, we further explore emerging strategies to enhance the activity of MSCs, including pretreatment and activation, lifestyle intervention, and genetic engineering techniques. This review provides a comprehensive framework for developing safer, standardized, and clinically transformative therapies based on MSCs.

## Graphical abstract

This schematic summarizes the major therapeutic mechanisms of MSCs across different disease systems and highlights factors associated with MSCs dysfunction, together with current strategies to enhance MSCs function and therapeutic efficacy.

This summary diagram illustrates that MSCs exert therapeutic effects in various diseases through multiple mechanisms, including multilineage differentiation, paracrine effects, immunomodulation, and mitochondrial transplantation. However, due to various factors, MSCs function may decline, and strategies such as pharmacological intervention, lifestyle modification, and genetic engineering can enhance MSCs function.

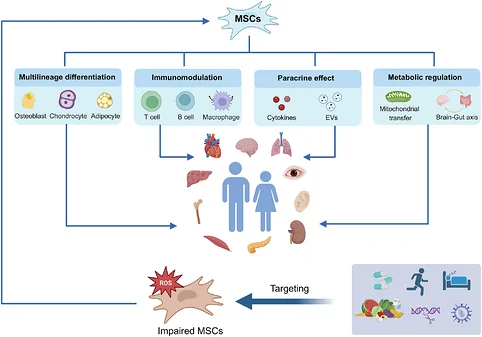

## 1 Introduction

With the increasing aging of the global population, chronic and degenerative diseases have emerged as major public health challenges worldwide [1]. Despite tremendous advances in modern medical technology, conventional pharmacological and surgical treatments often have limited capacity to restore the structure and function of damaged tissues. Consequently, regenerative medicine based on cell therapy has attracted considerable attention as a promising therapeutic strategy for a broad range of refractory diseases. Among the various cell types investigated, mesenchymal stromal cells (MSCs) have received particular interest because of their self‐renewal capacity, multilineage differentiation potential, immunomodulatory properties, and paracrine activity [2].

MSCs are multipotent stromal cells of mesodermal origin that can be isolated from adult tissues (e.g., bone marrow, adipose tissue, synovium, and dental pulp) as well as perinatal tissues (e.g., umbilical cord, umbilical cord blood, placenta, and amnion) [3]. Among these sources, bone marrow‐derived MSCs (BM‑MSCs), umbilical cord–derived MSCs (UC‑MSCs), and adipose tissue–derived MSCs (AD‑MSCs) are the most extensively investigated in clinical studies because robust protocols for their isolation and expansion have been established [4]. Meanwhile, MSCs derived from dental, oral, and craniofacial tissues have attracted increasing attention owing to their origin from highly dynamic remodeling niches, which may confer unique advantages for periodontal and craniofacial tissue regeneration [5, 6]. Under appropriate in vitro *or* in vivo induction conditions, MSCs can differentiate into mesodermal lineages, including adipocytes, chondrocytes, osteoblasts, and myocytes, and some studies have reported trans‐germ‐layer differentiation toward neural‐ or hepatocyte‐like phenotypes, although the physiological relevance of this plasticity remains under investigation [7]. MSCs exert therapeutic effects that are not solely dependent on direct differentiation into tissue‐specific cells. Instead, they interact dynamically with the injured or inflamed microenvironment through the secretion of growth factors (GFs), cytokines, and extracellular vesicles (EVs), as well as through direct cell–cell interactions, immune regulation, and mitochondrial or metabolic transfer to stressed cells [8, 9, 10]. Collectively, these mechanisms contribute to tissue repair, angiogenesis, immune regulation, metabolic homeostasis, and the resolution of inflammation. Coupled with their relative ease of isolation and expansion and their generally low immunogenicity, MSCs have therefore been extensively investigated for the treatment of tissue injury, hematological disorders, pulmonary fibrosis (PF), spinal cord injury (SCI), metabolic liver disease, optic nerve injury, and numerous other conditions [4, 10].

Despite these encouraging advances, several challenges continue to hinder the clinical translation of MSCs‐based therapies. Cellular senescence and functional deterioration can arise during donor aging or prolonged in vitro expansion, resulting in reduced proliferative capacity, altered secretory profiles, and diminished therapeutic efficacy [11]. This review provides a comprehensive overview of MSCs biology, therapeutic mechanisms, disease applications, and current challenges in clinical translation, while highlighting emerging strategies to enhance MSCs potency, thereby facilitating the development of standardized, high‐quality clinical‐grade MSCs products and broader clinical translation.

## 2 Biological Properties and Heterogeneity of MSCs

The biological properties of MSCs are highly heterogeneous and are shaped by multiple factors, including tissue origin, isolation strategy, culture conditions, and the surrounding microenvironment [12]. This intrinsic heterogeneity contributes to differences in their phenotype, proliferative capacity, differentiation potential, secretory profile, and immunomodulatory activity, ultimately influencing their therapeutic performance [13]. Accordingly, understanding the biological characteristics of MSCs is essential for the rational selection and clinical application of different MSCs populations.

### 2.1 Sources and Isolation

MSCs were first identified in the bone marrow and have subsequently been isolated from many other adult and perinatal tissues, including adipose tissue, synovial tissue, umbilical cord, umbilical cord blood, placenta, and amniotic membrane [14]. Among these, BM‐MSCs, UC‐MSCs, and AD‐MSCs remain the most extensively studied and clinically applied sources because of their accessibility, established isolation and expansion procedures, and extensive biological characterization [15, 16]. BM‐MSCs were originally described by Friedenstein and colleagues as plastic‐adherent fibroblast‐like cells capable of colony formation and osteogenic differentiation [17, 18]. However, their clinical application is limited by the invasive nature of bone marrow aspiration, restricted cell yield, and the age‐associated decline in proliferative and regenerative capacity [19]. By comparison, AD‐MSCs can be harvested in relatively large numbers through minimally invasive procedures and exhibit strong proliferative and immunomodulatory properties [20]. MSCs derived from perinatal tissues, particularly the umbilical cord and placenta, have also attracted considerable interest because these tissues are readily available after birth, raise few ethical concerns, and provide cells with high proliferative capacity, low immunogenicity, and suitability for large‐scale allogeneic production [21]. Beyond these major tissue sources, MSCs have also been isolated from dental, oral, and craniofacial tissues, including dental pulp MSCs (DP‐MSCs), periodontal ligament stromal cells (PDLSCs), gingiva‐derived MSCs (GMSCs), stromal cells from human exfoliated deciduous teeth (SHED), and cranial suture MSCs (SuSCs) [5, 22, 23]. These tissue‐specific MSCs populations have demonstrated promising regenerative potential, particularly in craniofacial reconstruction, periodontal regeneration, and bone repair [5, 23, 24, 25, 26]. However, compared with BM‐MSCs, AD‐MSCs, and UC‐MSCs, evidence for these emerging MSCs sources remains limited. In particular, SuSCs are still at an early stage of basic investigation, and further studies are needed to better define their biological characteristics and therapeutic potential [25].

Although MSCs derived from different tissues fulfill the core phenotypic criteria established by the International Society for Cell and Gene Therapy (ISCT), their biological properties are not identical. Increasing evidence indicates that tissue origin influences cell proliferation, differentiation capacity, secretory activity, and immunomodulatory function [27, 28]. These source‐dependent differences should therefore be taken into account when selecting MSCs for specific therapeutic applications.

### 2.2 Immunophenotype and Multipotency

**FIGURE 1:**
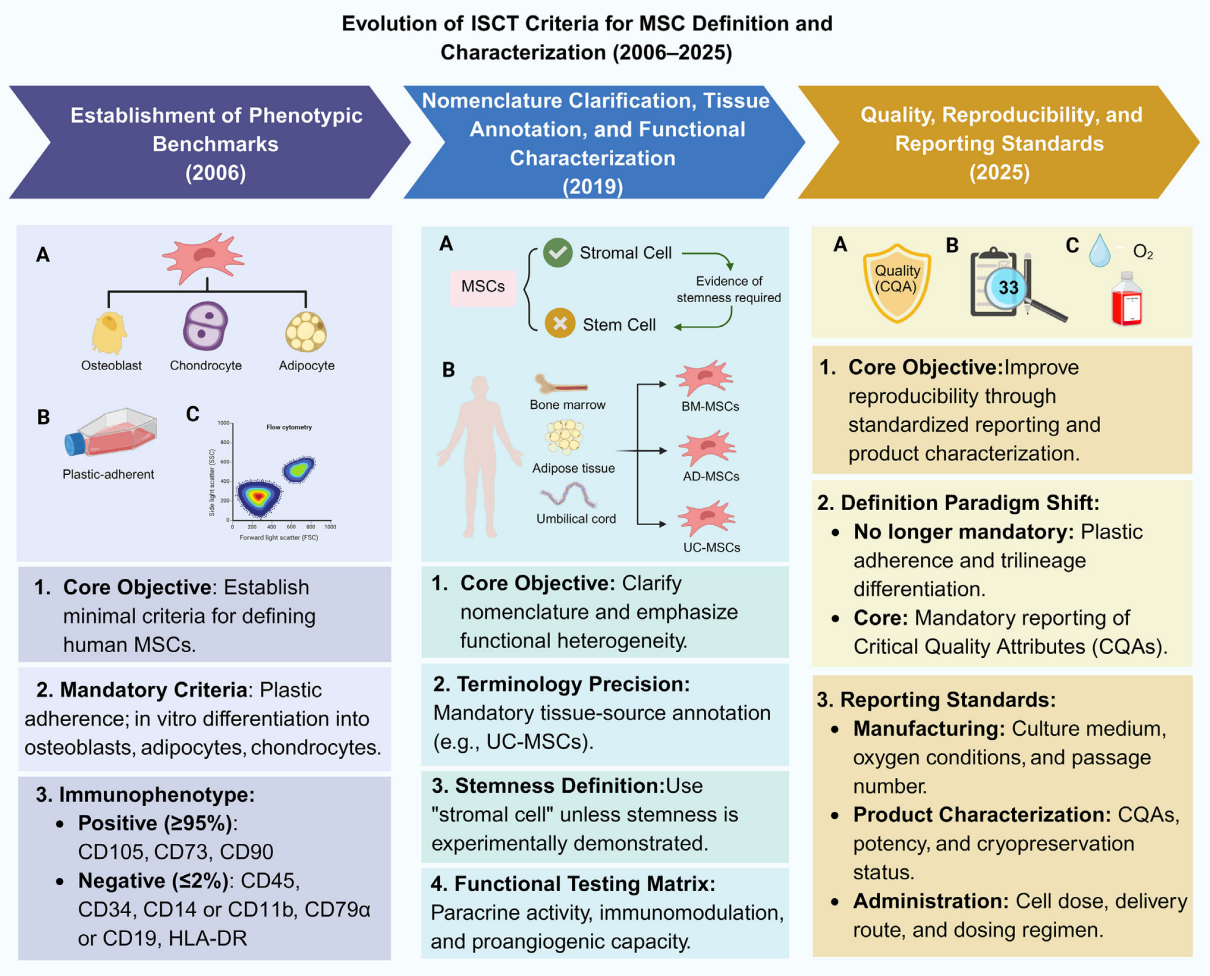
Evolution of ISCT criteria for MSCs definition and characterization: 2006–2025. The figure summarizes the progressive refinement of ISCT recommendations, from the establishment of minimal phenotypic criteria in 2006, through nomenclature clarification and functional characterization in 2019, to consensus‐based quality attributes and standardized reporting in 2025, reflecting the transition from cell definition toward reproducible clinical translation.

Immunophenotypic characteristics and multilineage differentiation potential constitute the principal biological features used to define MSCs. To harmonize MSCs characterization across studies, the ISCT proposed the minimal criteria for MSCs identification in 2006, which include plastic adherence under standard culture conditions, expression of CD73, CD90, and CD105, lack of expression of hematopoietic and immune‐related markers including CD34, CD45, CD14 or CD11b, CD79α or CD19, and HLA‐DR, and the capacity for osteogenic, chondrogenic, and adipogenic differentiation [27]. This characteristic surface marker profile distinguishes MSCs from hematopoietic and other stromal cell populations and has become the basis for their phenotypic identification. Recognizing the biological heterogeneity of MSCs, the ISCT subsequently recommended the term “mesenchymal stromal cells” in 2019 to more accurately describe these cells [29]. More recently, the updated ISCT framework published in 2025 placed greater emphasis on tissue origin, cell‐surface marker profiling, and functionally relevant potency assays, whereas plastic adherence and trilineage differentiation were no longer considered mandatory criteria for MSCs identification [30] (Figure 1).

Despite these refinements in nomenclature and characterization standards, multipotency remains a defining biological property of MSCs. Under appropriate induction conditions, they retain the capacity to differentiate into osteogenic, chondrogenic, and adipogenic lineages, which continues to serve as an important functional readout in basic and translational studies [31].

### 2.3 Paracrine Mechanisms and EVs

Paracrine signaling is now recognized as a major mechanism underlying the biological activity of MSCs. Rather than acting solely through direct differentiation, MSCs communicate with surrounding cells by releasing a broad repertoire of bioactive factors, including soluble proteins such as GFs and cytokines, as well as particulate mediators like EVs [32]. Among these secreted components, EVs have emerged as key mediators of MSCs function. EVs are lipid bilayer‐enclosed, nonreplicating extracellular particles that transport proteins, lipids, and diverse RNA species between cells [33]. Based on their biogenesis, EVs are generally categorized into exosomes (30–150 nm), which originate from multivesicular bodies; microvesicles (100–1000 nm), which are generated by outward budding of the plasma membrane; and apoptotic bodies (ABs; > 1000 nm, typically 1–5 µm), which are released during programmed cell death [34]. Although these vesicle populations show partial overlap in size, their classification is primarily based on their mechanisms of biogenesis. Through the transfer of bioactive cargoes, EVs modulate signaling pathways in recipient cells and mediate many of the immunomodulatory and regenerative functions of MSCs [35].

### 2.4 Immunomodulation

Immunomodulation is a defining biological property of MSCs and forms the basis for their broad therapeutic application [10, 36]. MSCs exhibit low immunogenicity, characterized by low‐to‐moderate expression of major histocompatibility complex class I (MHC‐I) molecules and minimal or absent expression of MHC‐II and costimulatory molecules, enabling their use in allogeneic transplantation with a relatively low risk of immune rejection [37]. Rather than exerting constitutive immunosuppression, MSCs dynamically adapt their immunoregulatory functions in response to the local inflammatory microenvironment. In highly inflammatory settings, MSCs primarily suppress excessive immune activation and promote inflammation resolution, whereas under low‐inflammatory conditions they help maintain immune homeostasis by supporting balanced immune responses [38]. MSCs modulate both innate and adaptive immunity through direct cell–cell interactions and the secretion of soluble mediators and EVs [39]. These coordinated actions shape the function of multiple immune cell populations, promoting the resolution of excessive inflammation while maintaining immune homeostasis. This context‐dependent immunoregulatory capacity is a hallmark of MSCs biology and provides the biological rationale for their therapeutic application across a broad spectrum of inflammatory, autoimmune, and degenerative disorders [40].

### 2.5 Source‐Dependent Functional Differences

The tissue source of MSCs is a major determinant of their biological properties and therapeutic potential. MSCs isolated from different tissues exhibit distinct proliferative capacities, secretory profiles, and immunomodulatory functions [28]. Proliferative potential varies markedly among MSCs sources and is strongly influenced by donor age. MSCs derived from perinatal tissues, such as the umbilical cord and placenta, are considered “zero‐year‐old” cells and display the greatest proliferative capacity [21, 41]. In contrast, both the frequency and proliferative activity of BM‐MSCs and AD‐MSCs decline progressively with aging [42, 43]. Expansion conditions further contribute to these differences. During early in vitro culture (Passages 1–5), AD‐MSCs exhibit higher proliferative activity and lower expression of senescence‐associated markers than BM‐MSCs [28].

Distinct tissue origins are likewise associated with differences in secretory activity. UC‐MSCs produce higher levels of multiple cytokines, including G‐CSF, GM‐CSF, HGF, IL‐6, IL‐8, and IL‐11, whereas BM‐MSCs express greater amounts of vascular endothelial growth factor (VEGF), suggesting a stronger proangiogenic potential [44, 45]. Although both BM‐MSCs and AD‐MSCs can promote angiogenesis in hindlimb ischemia models, AD‐MSCs have been reported to exhibit superior angiogenic capacity in some preclinical studies. Evidence regarding which cell source is clinically superior for therapeutic angiogenesis remains insufficient at present [46, 47]. Source‐dependent differences are also evident in hematopoietic support. While some studies have reported that umbilical cord blood‐derived MSCs (UCB‐MSCs) secrete higher levels of hematopoiesis‐related cytokines, others indicate that BM‐MSCs provide superior support for hematopoietic stem cell (HSC) colony formation [48, 49].

Immunomodulatory properties likewise vary according to tissue origin. Compared with BM‐MSCs, UC‐MSCs and AD‐MSCs generally exhibit lower immunogenicity and greater immunomodulatory activity [50, 51]. Under inflammatory stimulation with TNF‐α and IFN‐γ, HLA‐DR expression is upregulated in BM‐MSCs, whereas UC‐MSCs and certain other perinatal MSCs maintain relatively low HLA‐DR expression, indicating a potential advantage for allogeneic applications [52, 53]. In addition, AD‐MSCs more effectively regulate dendritic cell (DC) function than BM‐MSCs, as evidenced by enhanced IL‐10 secretion, reduced expression of CD80, CD86, and CD83, and stronger inhibition of DC differentiation and maturation [54]. Together, these findings underscore the substantial functional heterogeneity among MSCs from different tissue sources and support source selection according to the pathological characteristics and therapeutic requirements of specific diseases.

## 3 Mechanisms of Action in Disease Contexts

The therapeutic effects of MSCs are mediated by multiple, interconnected mechanisms, including immunomodulation and anti‐inflammation, tissue repair and regeneration, metabolic regulation, and homing and EV‐mediated intercellular communication [55]. This section aims to elucidate the common mechanisms by which MSCs exert their effects across multiple diseases.

### 3.1 Immunomodulation and Anti‐Inflammation

MSCs interact with innate and adaptive immune cells through direct cell–cell contact and the secretion of bioactive mediators, thereby modulating inflammatory responses and maintaining immune homeostasis [10]. Through these coordinated interactions, MSCs exert broad immunomodulatory effects that contribute to the treatment of inflammatory, autoimmune, and degenerative diseases [56].

MSCs regulate T‐cell responses by restoring the Th17/Treg balance, enhancing the suppressive function of regulatory T cells (Tregs), and promoting CD80–CTLA‐4‐mediated interactions that further strengthen Treg immunoregulatory activity [57, 58, 59]. For example, MSCs‐secreted HGF has been shown to partially mediate the restoration of the Th17/Treg balance by suppressing Th17 responses while promoting Treg expansion, thereby attenuating inflammatory responses [57]. In addition, MSCs transfer functional mitochondria to Tregs in an HLA‐dependent manner, which enhances their immunosuppressive activity [58]. The interaction between CD80 on MSCs and CTLA‐4 on Tregs further enhances the suppressive function of Tregs, thereby promoting immune tolerance [59]. Furthermore, GMSC‐derived EVs (GMSC‐EVs) promote the Treg/Th17 balance through inhibition of the IKKβ/NF‐κB pathway [60]. MSCs also modulate B‐cell responses. Human UC‑MSCs have been shown to promote the expansion of regulatory B cells (Bregs, specifically IL‐10+ B10 cells) through TGF‐β1‐dependent mechanisms [61]. Moreover, MSCs‑treated CD23^+^CD43^+^ Bregs secrete higher levels of IL‑10 while concurrently suppressing inflammatory T‑cell responses [62]. These findings highlight the role of MSCs in maintaining B‐cell immune tolerance through the expansion and functional enhancement of Breg subsets.

In addition to regulating lymphocyte responses, MSCs also modulate innate immune cells, particularly macrophages, neutrophils, and DCs, thereby contributing to inflammation resolution and immune homeostasis [63, 64, 65, 66]. For instance, AD‐MSCs restore the CX3CR1^+^ synovial lining macrophage barrier, accompanied by reduced expression of IL‐6, IL‐1β, and TNF‐α, and increased circulating IL‐10 levels, thereby alleviating synovial inflammation [63]. Similarly, exosomes derived from SHED promote macrophage polarize toward an M2 state, associated with upregulated anti‐inflammatory markers (CD206, Arg‐1, IL‐10, TGF‐β) and enhanced antioxidant activities (TAC, CAT, SOD), which may contribute to reduced inflammation and tissue injury [67]. Beyond macrophage regulation, MSCs also modulate neutrophils and DCs. GMSCs have been reported to reduce neutrophil infiltration and neutrophil extracellular trap (NET) formation through PGE2‐mediated activation of PKA and inhibition of ERK signaling, thereby limiting excessive inflammatory responses [65]. In addition, UC‐MSCs expand tolerogenic CD1c^+^ DC populations through FLT3L‐mediated signaling [66]. Moreover, AD‐MSCs‐derived EVs regulate DC function by transferring functional mitochondria, resulting in metabolic reprogramming and altered immune activity [68].

Overall, MSCs achieve immunomodulation by regulating multiple immune cell populations through diverse soluble factors and signaling pathways, including TGF‐β, PGE2, indoleamine 2,3‐dioxygenase (IDO), nitric oxide (NO), HGF, and IL‐10, thereby promoting inflammation resolution and immune homeostasis [57, 61, 62, 65, 69].

### 3.2 Tissue Repair and Regeneration

MSCs promote tissue repair and regeneration through multilineage differentiation, homing to sites of injury, and paracrine signaling mediated by secreted factors and EVs [70, 71].

MSCs possess multilineage differentiation capacity. Under appropriate induction conditions, they differentiate into mesoderm‐derived cell types, including osteoblasts, chondrocytes, adipocytes, and myocytes, and have also been reported to generate neural‐ and hepatic‐like cells under specific experimental conditions [72, 73]. This plasticity allows MSCs to contribute directly to the replacement of cells lost following injury or ageing. The homing effect is a critical prerequisite for MSCs to target and repair injured tissues. MSCs rely on chemokine–receptor axes, such as SDF‐1/CXCR4, to guide their directional migration toward injured or inflamed tissues [74]. Upon reaching the vascular endothelium, MSCs adhere to endothelial cells (ECs) through adhesion molecule‐mediated interactions, whereas subsequent transendothelial migration is facilitated by chemokine signaling and extracellular matrix (ECM) remodeling [75, 76].

In addition to direct cellular effects, MSCs promote tissue repair predominantly through paracrine signaling, particularly that mediated by EVs [77]. MSCs‐derived EVs promote target‐cell proliferation and migration, regulate ECM remodeling by balancing collagen synthesis and degradation, and stimulate angiogenesis through the delivery of proangiogenic proteins and regulatory miRNAs [77, 78, 79]. For example, miR‐146a attenuates endothelial senescence and promotes angiogenesis by inhibiting Src, whereas miR‐214‐3p and miR‐486 enhance endothelial migration and angiogenic activity through modulation of the PTEN/AKT pathway [80, 81, 82]. In addition, miR‐124 stabilizes the p62‐Keap1‐Nrf2 signaling axis, thereby enhancing autophagy, alleviating oxidative stress, and ultimately contributing to neuroprotection [83]. Soluble factors released by MSCs further support tissue regeneration. MSCs‐derived IGF‐2 restores the regenerative capacity of senescent chondrocytes, whereas VEGF, HGF, and IGF‐1 contribute to angiogenesis, cell survival, and tissue repair [84, 85]. Through these complementary mechanisms, MSCs facilitate structural and functional recovery across a broad range of tissues, including cartilage, skin, myocardium, and bone [86].

### 3.3 Metabolic Regulation

Beyond immunomodulation and tissue repair, MSCs also contribute to the maintenance of metabolic homeostasis by regulating glucose and lipid metabolism, mitochondrial function, and the gut microbiota [87, 88, 89, 90].

MSCs regulate glucose metabolic disorders by improving insulin sensitivity and promoting the restoration of pancreatic β‐cell function [91]. Under inflammatory stimulation, MSCs exhibit enhanced glycolytic activity, accompanied by the upregulation of glycolysis‐related genes such as PFKFB3 and HIF‐1α [87]. Conversely, HIF‐1α reinforces the immunomodulatory function of MSCs by promoting the secretion of immunosuppressive factors, including IDO, COX2, and PD‐L1 [92, 93]. MSCs improve lipid metabolism disorders by regulating the expression of lipid metabolism‐related genes and the activity of signaling pathways. Studies have shown that MSCs can reduce lipid accumulation in multiple tissues, including the liver and nervous tissue, by promoting fatty acid β‐oxidation and regulating cholesterol metabolism [88, 94, 95]. These effects are further reinforced by MSCs‐derived EVs, which deliver regulatory miRNAs to recipient cells. Among them, miR‐99b‐5p promotes autophagy through inhibition of mTOR signaling, miR‐24‐3p reduces lipid deposition by targeting Keap‐1, and miR‐627‐5p improves lipid metabolism through suppression of FTO expression [96, 97, 98]. Metabolic regulation by MSCs also involves the transfer of functional mitochondria to injured cells. Mitochondria can be delivered through tunneling nanotubes (TNTs), EVs or direct cell fusion, thereby contributing to the restoration of bioenergetic profiles in recipient cells [89, 99]. Studies have demonstrated that MSCs‐mediated mitochondrial transfer significantly enhances TCA cycle activity and promotes fatty acid synthesis, thereby facilitating EC proliferation and the release of proangiogenic factors [100].

Emerging evidence further suggests that modulation of the gut microbiota contributes to the metabolic benefits of MSCs therapy [90]. MSCs not only reshape the composition and diversity of intestinal microbial communities but also indirectly influence the gut microenvironment through EV‐mediated regulation of intestinal immunity and barrier integrity [90, 101]. MSCs‐derived miR‐181a enhances the expression of the tight junction proteins Claudin‐1 and ZO‐1, thereby promoting intestinal barrier function [102]. Together, these mechanisms allow MSCs to play important roles in treating metabolic, inflammatory, and immune‐mediated diseases.

## 4 Disease‐Specific Applications: Evidence From Preclinical and Clinical Studies

**FIGURE 2:**
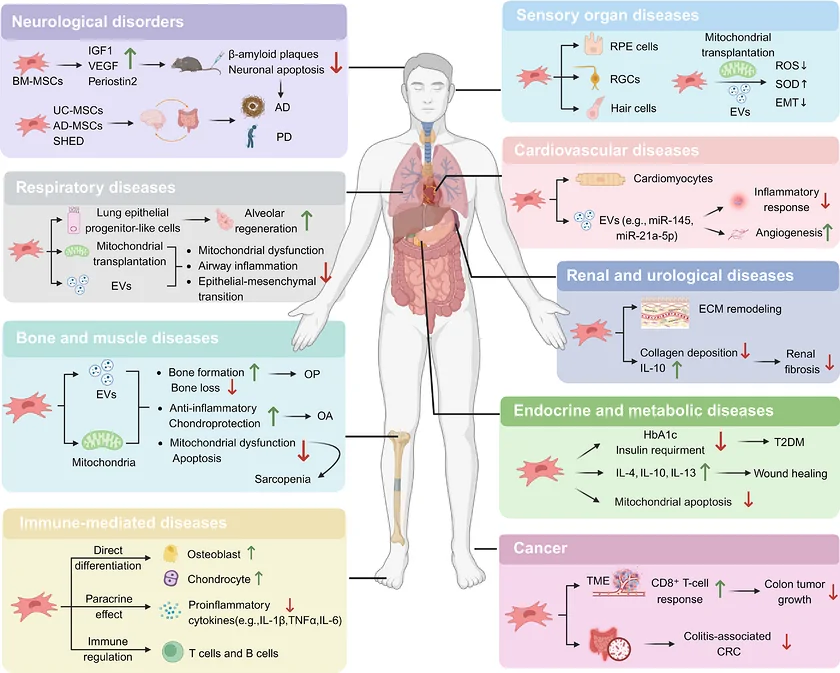
Therapeutic mechanisms and applications of MSCs across different disease systems. MSCs exert therapeutic effects in musculoskeletal, cardiovascular, immune‐mediated, neurological, respiratory, renal and urological, endocrine and metabolic, sensory organ diseases, and cancer through multiple mechanisms, including multilineage differentiation potential, immunomodulation, paracrine effects, and mitochondrial transfer.

The mechanisms discussed above provide a shared basis for understanding MSCs therapy. However, their relative importance varies across disease contexts, depending on the local pathological microenvironment. The following sections summarize representative preclinical evidence across major disease categories, illustrating how these common mechanisms translate into disease‐specific therapeutic effects (Table 1 and Figure 2).

**TABLE 1:** Preclinical therapeutic applications of MSCs in animal disease models.

Disease category	MSCs source	Animal model	Route of administration	Major findings	References
KOA	UC‐MSCs‐derived EVs	Mouse	IAI	Induce hyaline cartilage regeneration.	[103]
OA	AD‐MSCs	Canine	IAI	Anti‐inflammatory and cartilage protection.	[104]
Sarcopenia	UC‐MSCs‐derived EVs	Mouse	Tail‐vein injection	Ameliorate skeletal muscle atrophy and dysfunction.	[105]
MRONJ	AD‐MSCs‐derived EVs	Mouse	Local injection	Reduce inflammation accelerate gingival wound healing and tooth socket bone regeneration.	[106]
Intervertebral disk degeneration	PRP combined with AD‐MSCs	Rabbit	Intradiscal injection	COL2A1i, COL10A1sc	[107]
HLI	AD‐MSCs	Rat	Intramuscular injection	Improve blood flow and promote the formation of new blood vessels.	[46]
MI	BM‐MSCs‐derived ABs	Rat	IMI	Enhance angiogenesis and improve cardiac functional recovery.	[108]
	UC‐MSCs (maternal segment)	Rat	IMI	Reduce myocardial cell apoptosis and promote angiogenesis.	[109]
Chronic ICM	BM‐MSCs	Porcine	IV	Reduce myocardial hypertrophy and fibrosis.	[110]
RA	AD‐MSCs‐derived EVs	Rat	Tail‐vein injection	Suppresses arthritis by promoting M2 polarization and suppressing glycolysis.	[111]
SLE	BM‐MSCs	Mouse	Tail‐vein injection	Regulate immune homeostasis.	[112]
	UC‐MSCs	Mouse	Tail‐vein injection	Ameliorate SLE through modulating gut microbiota–tryptophan–AHR axis.	[113]
AD	UC‐MSCs, SHED, AD‐MSCs	Mouse	IV	Exert neuroprotection via the microbiome–gut–brain axis.	[114]
Perinatal brain injury	BM‐MSCs	Rat	IV	Improve sensorimotor and cognitive function, and enhance neuronal growth.	[115]
ICH	UC‐MSCs	Mouse	IV	Robust neuroprotection through coordinated local and systemic immunomodulation.	[116]
COPD	BM‐MSCs/BM‐MSCs‐derived EVs	Mouse	IV/ Intratracheal instillation	Reduce lung inflammation, apoptosis, EMT, and collagen deposition.	[117]
PF	BM‐MSCs	Mouse	Intratracheal instillation	Inhibit the expression of ZC3H4 in macrophages to alleviate inflammation and fibrosis.	[118]
IPS	BM‐MSCs	Mouse	Tail‐vein injection	Induce the polarization of M2 macrophages and promote the formation of regulatory CCR2+ CD4+ T cells.	[119]
ARDS	UC‐MSCs	Mouse	Tail‐vein injection	Reduce pulmonary inflammation and attenuate lung injury.	[120]
Kidney injury and fibrosis	BM‐MSCs	Rat	Tail‐vein injection	Boost antioxidant defense and reduce apoptosis and inflammation.	[121]
AKI	BM‐MSCs and UC‐MSCs	Mouse	Tail‐vein injection	Inhibit the activation of NLRP3 inflammasome and reduce the deposition of CaOx crystals.	[122]
IC/BPS	BM‐MSCs‐derived EVs	Mouse	local injection (Bladder wall)	Enhance tissue healing and anti‐inflammatory capabilities.	[123]
Colon cancer	BM‐MSCs‐derived exosomes	Mouse	Tail‐vein injection	Advance anti‐PD‐1 therapy by modulating tumor immune environment.	[124]
CAC	Intestinal MSCs	Mouse	Intraperitoneal injection	Ameliorate colitis and partially revert intestinal dysbiosis.	[125]
BCP	UC‐MSCs	Rat	Intrathecal injection	Inhibit the excessive activation of spinal dorsal horn neurons and glial cells.	[126]
NASH	UC‐MSCs‐derived exosomes	Mouse	Tail‐vein injection	Regulate the anti‐inflammatory phenotype of macrophages.	[127]
Corneal injury	BM‐MSCs	Mouse	Intrastromal and subconjunctival injection	Promote wound healing, reduce angiogenesis and turbidity phenomena	[128]

Abbreviations: CAC, colitis‐associated colorectal cancer; HLI, hindlimb ischemia; IMI, intramyocardial injection; IV, intravenous infusion; KOA, knee osteoarthritis; NASH, nonalcoholic steatohepatitis; PRP, platelet‐rich plasma.

### 4.1 Bone and Muscle Diseases

Bone and muscle diseases, including osteoporosis (OP), osteoarthritis (OA), and sarcopenia, are characterized by progressive disruption of tissue homeostasis and impaired regenerative capacity [129]. In preclinical models, MSCs‐based therapies attenuate disease progression by modulating the inflammatory microenvironment, promoting osteochondral regeneration, and restoring cellular metabolic homeostasis [130, 131].

OP is a metabolic bone disease characterized by decreased bone mass and disrupted bone microarchitecture [132]. Transplanted BM‐MSCs undergo apoptosis within 2 days postinjection and release apoptotic EVs enriched with Ras protein. Upon phagocytosis by endogenous BM‐MSCs, these EVs activate the Ras/Raf1/Mek/ERK signaling pathway, thereby reducing bone loss and promoting bone formation in OP mouse models [133]. Additionally, macrophages in the osteoporotic microenvironment release oxidatively damaged mitochondria that are taken up by MSCs, impairing their osteogenic differentiation, suggesting that targeting this mitochondrial transfer represents a potential therapeutic approach for OP [132]. Genetic silencing further enhances MSCs osteogenic potential. Smurf1‑silenced MSCs exhibit enhanced osteogenic capacity and antiresorptive activity through paracrine effects, significantly promoting bone formation while reducing bone loss [134].

OA is characterized by progressive cartilage degeneration driven by an imbalance between anabolic and catabolic activities within chondrocytes [135]. Most therapeutic effects of MSCs in OA are mediated by their derived EVs. For example, exosomes derived from subcutaneous AD‐MSCs deliver miR‐199a‐3p to chondrocytes, playing an anticatabolic role and improving cartilage repair in DMM‐induced OA rats [136]. Engineered IL‑6‑enriched MSCs‑derived EVs suppress cartilage degradation and synovitis through the mt‑ND3/NADH‐CoQ axis, decelerating the progression of OA [137]. In addition to EV‐mediated effects, UC‐MSCs transfer mitochondria to OA chondrocytes, restoring redox balance and cellular bioenergetics, which may contribute to maintaining ECM homeostasis [138]. This mechanism has been validated in temporomandibular joint osteoarthritis (TMJOA), where AD‐MSCs‐derived EVs transfer functional mitochondria to DCs, activating the ERK1/2‐FoxO1‐autophagy pathway and reshaping their metabolic state, thereby alleviating inflammation [68]. The chondroprotective and anti‑inflammatory effects of UC‑MSCs‑derived EVs and AD‐MSCs have been validated in different models of OA [103, 104, 139]. Compared with intra‑articular injection (IAI), subchondral (SC) delivery of human UC‑MSCs has been shown to more effectively maintain SC bone microarchitecture in OA models [140]. Beyond structural protection, MSCs and their derived EVs also alleviate OA‑associated pain. In a DMM‑induced murine OA model, this analgesic effect was attributed to the prevention of excessive dorsal root ganglion (DRG) neuronal excitation, as treated mice showed pain behaviors similar to sham‐operated mice (compared with untreated controls) [141].

Sarcopenia is a systemic skeletal muscle disorder characterized by the progressive loss of muscle mass and function, primarily driven by cellular senescence and mitochondrial dysfunction [142]. UC‐MSCs activate autophagy and downregulate the p16/p53‐p21 axis to delay myocyte aging [143]. Both UC‐MSCs and their derived EVs promote mitochondrial biogenesis and suppress apoptosis in muscle cells. In SAMP10 accelerated‐aging mice, tail‐vein injection of UC‐MSCs or their derived EVs improved grip strength and exercise endurance, increased muscle fiber cross‐sectional area, and ameliorated mitochondrial ultrastructure [105, 144]. Combined exercise and AD‐MSCs administration showed superior effects over either monotherapy, suggesting that lifestyle interventions and cell‐based therapies may exert synergistic effects [145]. In addition, AD‐MSCs‐derived EVs have shown therapeutic potential in preventing medication‐related osteonecrosis of the jaw (MRONJ), while MSCs‐based therapies have been evaluated in intervertebral disk degeneration models [106, 107]. Overall, these preclinical evidences strongly support the therapeutic potential of MSCs in musculoskeletal disorders through coordinated regulation of osteogenesis, chondroprotection, and muscle homeostasis.

### 4.2 Cardiovascular Diseases

Cardiovascular diseases, including myocardial infarction (MI), heart failure (HF), and atherosclerosis (AS), are major causes of morbidity and mortality worldwide [146]. In preclinical studies, MSCs promote cardiac and vascular repair through coordinated regulation of inflammation, angiogenesis, cell survival, and tissue remodeling [147].

MI is characterized by extensive cardiomyocyte death and microvascular destruction following coronary artery occlusion [148]. MSCs primarily exert myocardial protection by inhibiting inflammation, promoting angiogenesis, reducing fibrosis, and enhancing cardiomyocyte survival [149]. When treated with 5‐azacytidine, both BM‐MSCs and AD‐MSCs can differentiate into cardiomyocyte‐like cells in vitro [150]. However, these differentiated cells remain functionally immature, suggesting that direct cardiomyocyte replacement is unlikely to account for the therapeutic benefits observed in vivo [151]. Instead, BM‐MSCs‐derived ABs promoteTFEB‐mediated autophagy to enhance angiogenesis and improve cardiac functional recovey after MI [108]. The therapeutic efficacy in these models is highly sensitive to cell‐source heterogeneity. Specifically, subsegmental analysis revealed that human UC‐MSCs from the maternal segment exhibited significantly superior capacity to restore cardiac function in rats compared with those from the middle or fetal segments [109]. Furthermore, the efficacy of MSCs has also been verified in a porcine model of chronic ischemic cardiomyopathy (ICM) induced by MI. However, this effect is dose‐dependent, and a three‐dose administration regimen is effective while a single dose is ineffective [110].

AS is driven by endothelial dysfunction, chronic vascular inflammation, and lipid accumulation within the arterial wall [152]. Increasing evidence indicates that MSCs‑derived EVs attenuate systemic inflammatory responses, restore angiogenesis and oxidative stress‐impaired migration of aged ECs, and improve endothelial function [78, 153]. AD‑MSCs‑derived EVs carrying miR‑146a inhibit Src phosphorylation, thereby modulating the phosphorylation status of vascular endothelial cadherin (VE‑cadherin) and Caveolin‑1 to reduce EC senescence and promote angiogenesis [80]. Additionally, AD‐MSCs‐derived EVs protect ECs against AS through miR‐342‐5p mediation [154]. However, Yang et al. found that miR‐145 derived from EVs of human UC‐MSCs reduces atherosclerotic plaque formation by inhibiting the migration of human umbilical vein endothelial cells (HUVECs) [155]. These findings suggest that MSCs from different sources may target distinct stages of atherogenesis. In contrast, BM‐MSCs‐derived EVs primarily modulate macrophage function. For example, BM‐MSCs‐derived exosomes carrying miR‐21a‐5p target KLF6/ERK1/2 pathways to promote anti‐inflammatory (M2) macrophage polarization, thereby reducing plaque formation and attenuating AS progression [156]. Similarly, exosome‑mediated delivery of si‑LOC100129516 promotes cholesterol efflux and suppresses lipid accumulation by activating the PPARγ/LXRα/ABCA1 signaling pathway, thereby alleviating AS progression, further highlighting the source‐dependent heterogeneity of MSCs‐derived EV therapeutic actions [157].

HF represents the common outcome of diverse cardiovascular disorders and is characterized by progressive ventricular remodeling and impaired cardiac function [158]. BM‑MSCs‑derived miR‑129‑5p targets tumor necrosis factor receptor‑associated factor 3 (TRAF3), attenuates NF‑κB signaling, and consequently alleviates ventricular dysfunction, oxidative stress, apoptosis, inflammation, and fibrosis in experimental HF [159]. However, there is currently no consensus on the optimal EV source or administration route for clinical application.

### 4.3 Immune‐Mediated Diseases

Immune‐mediated diseases, including rheumatoid arthritis (RA) and systemic lupus erythematosus (SLE), are characterized by immune dysregulation that drives chronic inflammation and autoimmune tissue damage [160]. RA is a prototypical autoimmune disease characterized by chronic joint inflammation and bone erosion, largely associated with Th17/Treg imbalance and excessive production of proinflammatory cytokines [161]. Although conventional therapies, including nonsteroidal anti‐inflammatory drugs (NSAIDs), glucocorticoids (GCs), and biologics, can effectively control disease activity, their long‐term use is frequently associated with adverse effects [162]. MSCs suppress inflammation and promote tissue repair in RA through their immunomodulatory capacity and regenerative potential. They inhibit osteoclastogenesis and reduce proinflammatory cytokine production via paracrine signaling, thereby limiting bone erosion and cartilage destruction [163]. Their osteogenic and chondrogenic potential may also contribute to the regeneration of damaged joint tissues [164]. The therapeutic efficacy of MSCs in RA has been extensively validated in animal models, particularly through the application of MSCs‑derived EVs. MSCs‐derived microvesicles downregulate IL‐1β, TNF‐α, IL‐6 in peripheral blood and IL‐1β, TNF‐α, MMP‐13, and ADAMTS‐5 in cartilage, thereby inhibiting inflammation‐induced cartilage degradation in collagen‑induced arthritis (CIA) mouse models [165]. The therapeutic efficacy of MSCs‐derived EVs can also be enhanced through combinatorial approaches. Icariin (ICA), an anti‐inflammatory compound isolated from Epimedium, loaded into AD‐MSCs‐derived exosomes, suppressed glycolytic metabolism by inhibiting the ERK/HIF‐1α/GLUT1 pathway, promoted macrophage M2 polarization, and ameliorated synovitis and cartilage erosion in CIA rats [111].

SLE is a chronic multisystem autoimmune disease characterized by autoantibody production and immune complex deposition [166]. MSCs exert therapeutic effects in SLE by modulating T cells, B cells, macrophages, and DCs. Recent studies have shown that MSCs restore immune homeostasis by suppressing pathogenic T‐cell responses, inhibiting B‐cell activation and differentiation, promoting Breg expansion, inducing tolerogenic DCs, and promoting M2‑type macrophage polarization [66, 112, 167, 168]. In SLE mice, BM‐MSCs transplantation triggers neutrophil aggregation and ICAM‐1/Rab11b‐mediated EV release, and restores the Th17/Treg balance via the LILRB4/STAT5/STAT3 axis, thereby delaying the progression of SLE [112]. Moreover, human UC‐MSCs alleviate SLE progression through modulation of gut microbiota dysbiosis in MRL/lpr spontaneous lupus mice [113]. However, the clinical safety and long‐term risks of MSCs therapy still require further evaluation through rigorous clinical validation in immune‐mediated diseases.

### 4.4 Neurological Disorders

Alzheimer's disease (AD), Parkinson's disease (PD), Huntington's disease (HD), and amyotrophic lateral sclerosis (ALS) are characterized by progressive neuronal loss and age‐related functional decline, and their incidence increases markedly with age [169]. Given the limited regenerative capacity of the central nervous system and the modest efficacy of conventional pharmacotherapies, MSCs‐based therapies have emerged as a potential strategy to modulate neuroinflammation, promote neural repair, and slow disease progression in neurodegenerative diseases [170].

The hallmark pathological features of AD include amyloid‑β (Aβ) deposition and tau aggregation, leading to neurodegeneration and consequent neuropsychiatric symptoms and cognitive decline [171]. MSCs and their derived EVs ameliorate AD pathology primarily by modulating local immune homeostasis, promoting clearance of pathological protein aggregates, and facilitating neuroprotection and regeneration [172]. BM‑MSCs secrete IGF‐1, VEGF, and Periostin2 to activate the AKT/IAPs signaling pathway, reducing Aβ plaque deposition and neuronal apoptosis [173]. Similarly, MSCs‐derived EVs recapitulate the therapeutic effects of their parental cells. In a mouse model carrying five familial AD mutations (5XFAD), intranasal administration of human MSCs‐derived EVs attenuated neuroinflammation, decreased Aβ plaque deposition, and improved cognitive function, thereby delaying disease progression [174]. Moreover, UC‐MSCs, SHED, and AD‐MSCs exert neuroprotective effects in AD mouse models through the microbiota–gut–brain axis, highlighting gut microbiota regulation as an emerging mechanism in MSCs‐mediated neuroprotection [114].

PD is a progressive neurodegenerative disorder characterized by the selective loss of nigrostriatal dopaminergic neurons and the presence of Lewy bodies [175]. Clinically, it presents with the classic motor triad of bradykinesia, rigidity, and resting tremor, while postural instability typically emerges in the later stages [176]. In contrast to AD, MSCs‐based treatment strategies for PD have focused primarily on cell replacement and immunomodulation. Human UC‐MSCs can be chemically induced to differentiate into dopaminergic neurons, which subsequently increases dopamine levels in the striatum and ameliorates motor deficits in PD mouse models [177]. This therapeutic strategy has been validated in toxin‐induced PD models. In MPTP‐induced PD mice, intrastriatal transplantation of BM‐MSCs improved motor function and promoted the survival of dopaminergic neurons in the substantia nigra [178]. Recent studies have suggested that MSCs‐based therapies may interact with the gut microbiota. MSCs can exert neuroprotective effects in AD and PD through the gut–brain axis, offering novel mechanistic insights and intervention strategies for treating neurodegenerative diseases [114, 179]. Additionally, the therapeutic effects of MSCs in nervous system injury and intracerebral hemorrhage (ICH) have been further validated in preclinical models [115, 116].

Although extensive preclinical studies support the therapeutic potential of MSCs in neurological disorders, the current evidence remains limited. The therapeutic efficacy varies considerably across different cell sources (e.g., bone marrow, adipose tissue, and umbilical cord) and administration routes (e.g., intravenous, intrathecal, and intracerebral). In particular, existing animal models, such as the MPTP‐induced PD model and the 5XFAD transgenic AD model, fail to capture the full complexity of human neurodegeneration, a limitation that further compromises their clinical translation.

### 4.5 Respiratory Diseases

Chronic respiratory diseases, particularly chronic obstructive pulmonary disease (COPD) and idiopathic pulmonary fibrosis (IPF), are characterized by progressive and largely irreversible structural damage to the lung [180]. COPD is a chronic inflammatory lung disease with persistent airflow limitation, encompassing two classical pathological phenotypes, chronic bronchitis and emphysema. The latter involves alveolar wall destruction and loss of elastic recoil [181]. In contrast, IPF is dominated by progressive pulmonary interstitial fibrosis, with core pathology involving myofibroblast activation and aberrant ECM remodeling with excessive deposition [182]. Despite these pathological differences, both conditions are associated with impaired alveolar regeneration and dysregulated immune responses.

In COPD, MSCs‑based approaches have been extensively explored for their ability to restore epithelial integrity and attenuate chronic inflammation. Lung‑derived MSCs (LMSCs) may have greater regenerative capacity than BM‐MSCs and AD‐MSCs, because they express high levels of GFs, including fibroblast growth factor 10 (FGF10) and HGF [183]. However, LMSCs derived from patients with COPD exhibit secretory defects that may undermine alveolar repair, highlighting a critical limitation of autologous LMSC‑based therapies [184]. In addition to tissue‑resident MSCs, UC‑MSCs and amniotic fluid MSCs (AF‑MSCs) can enhance alveolar repair. Intratracheal administration of AF‐MSCs predifferentiated into lung epithelial progenitor‐like cells effectively promotes alveolar regeneration and reverses emphysema [185]. UC‐MSCs‐based therapy has also entered clinical evaluation in patients with COPD, showing acceptable safety and early signs of efficacy [186]. MSCs‐derived EVs represent a promising cell‐free strategy for COPD. They exert protective effects on the lung by attenuating epithelial injury, suppressing mucus hypersecretion, and preserving alveolar structural integrity. For example, human UC‐MSCs‐derived EVs attenuate lung inflammation, decrease goblet‐cell numbers, and improve emphysematous lesions, whereas BM‐MSCs‐derived EVs reduce pulmonary microvascular endothelial‐cell apoptosis via the miR‐30b/Wnt5a signaling pathway [187, 188]. In cigarette‐smoke/lipopolysaccharide‐induced COPD mouse models, intravenous administration of UC‐MSCs‐derived EVs reduced inflammatory‐cell infiltration in bronchoalveolar lavage fluid (BALF) and attenuated alveolar destruction [189]. The optimal delivery route may vary with disease stage. Intravenous BM‐MSCs infusion more effectively suppressed inflammation during early‐stage COPD, whereas intratracheal administration was more beneficial in advanced emphysema [117]. Beyond pulmonary protection, MSCs‐derived EVs may have potential to ameliorate COPD‐associated neurocognitive disorders (COPD‐NCDs) [190]. Combining MSCs with antioxidants or anti‐inflammatory agents may further enhance efficacy, suggesting a potential role for combination therapy in slowing COPD progression [191].

In IPF, MSCs‐based therapies primarily aim to suppress fibroblast activation, limit ECM accumulation, and restore immune homeostasis [192]. In silica‑induced PF mouse models, repeated intratracheal administration of BM‑MSCs improved pulmonary retention and enhanced immunomodulatory function through the downregulation of ZC3H4, thereby more effectively suppressing PF [118]. Compared with placenta‐derived MSCs (PL‐MSCs), UC‐MSCs exhibit stronger antifibrotic activity, largely because of their superior capacity to induce M2 macrophage polarization [193]. Nevertheless, therapeutic efficacy is strongly influenced by the route of administration. Intravenous delivery of human UC‐MSCs has been reported to provide greater protection than intratracheal administration, partly by inhibiting the differentiation of Ly6C^+^ classical monocytes into M2 macrophages [194]. In addition to whole‐cell therapy, mitochondrial transplantation has emerged as a promising strategy for IPF. Transplantation of mitochondria isolated from human UC‐MSCs (PN‐101) alleviates IPF by inhibiting ECM protein deposition in lung fibroblasts and suppressing epithelial–mesenchymal transition (EMT) in lung epithelial cells [195]. However, several factors still affect therapeutic efficacy. For example, the antifibrotic activity of BM‐MSCs‐derived EVs is inverse dose‐dependent and appears strongest in early‐to‐moderate IPF rather than advanced disease, indicating that dosage and disease stage both matter for treatment outcomes [196]. These findings highlight the importance of optimizing cell source, delivery route, and treatment timing in IPF. In addition, MSCs have demonstrated therapeutic potential in several other respiratory disorders, including idiopathic pneumonia syndrome (IPS), chronic asthma, and acute respiratory distress syndrome (ARDS) [119, 120, 197].

### 4.6 Other Diseases

In addition to the major disease categories discussed above, MSCs have also been investigated in preclinical studies of renal and urological disorders, endocrine and metabolic diseases, sensory organ diseases, and cancers. Although evidence in these areas remains limited, the available studies broaden the potential therapeutic scope of MSCs‐based interventions.

#### 4.6.1 Renal and Urological Diseases

Diabetic kidney disease (DKD), acute kidney injury (AKI), and chronic renal failure (CRF) are common renal disorders, whereas urinary incontinence (UI) and benign prostatic hyperplasia (BPH) are prevalent lower urinary tract disorders [198, 199, 200, 201, 202]. Studies suggest that MSCs and their derived EVs may act in renal and urological diseases by attenuating inflammation, suppressing fibrosis and inflammasome activation, and facilitating ECM remodeling and tissue repair [96, 121, 122, 123, 198, 203, 204, 205, 206, 207, 208, 209, 210, 211, 212]. For example, human UC‐MSCs‐derived exosomes ameliorated renal injury by inhibiting NOD2‐driven inflammation, thereby reducing albuminuria and preserving renal function in a streptozotocin (STZ)‐induced DKD mouse model [213]. Likewise, both BM‐MSCs and UC‐MSCs reduced calcium oxalate crystal (CaOx) deposition and renal injury in AKI models by inhibiting NLRP3 inflammasome activation [122]. MSCs‐derived EVs also promote tissue repair, attenuate inflammation, and modulate immune responses in bladder disorders, including interstitial cystitis/bladder pain syndrome (IC/BPS) and radiation cystitis (RC) [123, 212]. Current MSCs‐based therapeutic research in prostate diseases has focused mainly on prostate cancer and prostatitis, whereas the potential role of MSCs in BPH remains largely unexplored [214, 215, 216]. Overall, the evidence supports anti‐inflammatory and reparative roles for MSCs in renal and urological diseases. However, most of this evidence comes from small‐animal models, and whether these effects produce durable functional recovery in chronic disease remains unclear.

#### 4.6.2 Endocrine and Metabolic Diseases

Diabetes mellitus (DM) and hypothyroidism are the most common endocrine diseases, and obesity is the most prevalent metabolic disorder [217, 218]. DM is characterized by impaired glucose homeostasis and progressive metabolic dysfunction [219]. MSCs exert their therapeutic effects in DM primarily through immunomodulation, promotion of pancreatic β‐cell regeneration, and improvement of insulin sensitivity, thereby improving glycemic control and ameliorating diabetic complications [220, 221, 222]. In diabetic foot ulcers (DFUs), perinatal tissue‐derived MSCs accelerate wound healing via activation of the PI3K/AKT pathway, while gene‐modified UC‐MSCs overexpressing anti‐inflammatory cytokines (IL‐4, IL‐10, IL‐13) enhance repair through macrophage polarization [223, 224]. Hypothyroidism is another common endocrine disorder with multisystem involvement [225]. Current MSCs therapies remain limited to preclinical models. For example, human amniotic membrane‐derived MSCs (AM‐MSCs) improved thyroid function via apoptosis inhibition in aged mice, and BM‐MSCs‐conditioned medium (CM) enhanced fracture healing in hypothyroidism rats [226, 227]. In obesity, MSCs have shown promise in ameliorating metabolic complications such as insulin resistance and hepatic steatosis [228, 229, 230]. Gene‐modified or pretreated MSCs enhance anti‐inflammatory and metabolic functions in obesity therapy [231]. Repeated administrations of IL10‐overexpressing UC‐MSCs reduced high‐fat diet‐induced obesity [230]. Importantly, despite the potential of these cells to treat obesity‐related complications, the efficacy of MSCs therapy in obesity is constrained by the obesogenic microenvironment. Obesity impairs the biological properties of autologous MSCs, including AD‐MSCs and BM‐MSCs, suggesting that allogeneic MSCs may represent a more suitable therapeutic option [229, 232].

#### 4.6.3 Sensory Organ Diseases

MSCs have been investigated in several sensory disorders, including retinal degeneration, glaucoma, cataracts, and age‐related hearing loss (ARHL) [231].

In retinal diseases, particularly age‐related macular degeneration (AMD), MSCs promote retinal repair through both differentiation and paracrine effects. Human UC‐MSCs can be induced to differentiate into retinal pigment epithelial (RPE) cells in vitro and subsequently transplanted for the treatment of retinal degeneration [233]. UC‐MSCs‐derived exosomes can attenuate subretinal fibrosis by inhibiting EMT in RPE cells [234]. Additionally, mitochondrial transfer from AD‐MSCs and WJ‐MSCs to RPE cells reduces oxidative stress and enhances cell viability [235, 236]. Engineered MSCs‐derived EVs also show therapeutic potential for retinal disease. Pigment epithelium‐derived factor (PEDF)‐loaded MSCs‐derived small EVs enhance PEDF delivery stability and exhibit antiangiogenic, anti‐inflammatory, and neuroprotective effects to suppress pathological retinal neovascularization in oxygen‐induced retinopathy models [237]. In addition, composite engineered exosomes designed for combination therapy with anti‐VEGF agents have shown potential to suppress wet AMD [238]. Glaucoma is a progressive neurodegenerative disease of the optic nerve characterized by retinal ganglion cell (RGC) loss, optic nerve degeneration, and progressive visual field impairment, with elevated intraocular pressure (IOP) being the major modifiable risk factor [236, 239]. BM‐MSCs can differentiate into RGCs in the vitreous cavity of a glaucoma rat model and exhibit potential for neuroprotection and neuroregeneration [240]. Several studies indicate that the MSCs‐derived secretome promotes RGC survival and protects the trabecular meshwork in glaucoma models [241, 242, 243, 244]. For example, human AM‐MSCs‐derived exosome‐rich conditioned medium (AMSC‐ERCM), which is abundant in GFs and neurotrophic factors (NFs), can reduce IOP, protect retinal cells, and reverse retinal layer atrophy, thereby presenting a promising therapeutic strategy for glaucoma [241]. UC‐MSCs‐derived EVs can alleviate optic nerve damage caused by chronic high IOP by inhibiting apoptosis [242]. BM‐MSCs‐derived CM rescues TM cells from oxidative stress‐associated autophagy and apoptosis, while their EVs attenuate TGF‐β2‐induced TM fibrosis and reduce IOP elevation in vivo [243, 244]. In cataracts, human UC‐MSCs‐derived exosomes can inhibit EMT in lens epithelial cells (LECs), thereby delaying the progression of fibrotic cataracts [245, 246].

MSCs have also shown therapeutic potential in ARHL by promoting cochlear hair cell regeneration and facilitating functional hearing recovery [247, 248, 249, 250]. Collectively, these findings highlight the broad applicability of MSCs‐based therapies for sensory organ diseases. However, the available evidence remains limited, and further studies are needed to better define their therapeutic potential.

#### 4.6.4 Cancer

MSCs have shown therapeutic potential in cancer through modulation of the tumor microenvironment (TME) [251]. BM‐MSCs‐derived miR‐125b‐5p reduced regulatory T‐cell infiltration and enhanced CD8^+^ T‐cell responses in colon cancer, whereas intestinal MSCs alleviated colitis‐associated colorectal cancer by suppressing inflammation and ameliorating gut dysbiosis [124, 125]. MSCs have also demonstrated value in supportive care by alleviating bone cancer pain (BCP), and IL‐6‐deficient MSCs enhanced the sensitivity of acute myeloid leukemia (AML) cells to cytarabine (Ara‐C) [126, 252]. Nevertheless, accumulating evidence indicates that MSCs may also facilitate tumor progression by promoting metastasis, metabolic adaptation, and therapeutic resistance under specific microenvironmental conditions [253, 254, 255]. These findings highlight that the therapeutic outcome of MSCs in cancer is highly context‐dependent and requires careful optimization of cell source and therapeutic strategy.

In conclusion, current preclinical evidence indicates that MSCs have therapeutic potential across diverse disease models, although the dominant mode of action varies according to disease pathology and the local microenvironment. Immunomodulation is prominent in immune‐mediated diseases, whereas tissue repair and regeneration are central in musculoskeletal and neurological disorders [136, 163, 172]. In diseases associated with mitochondrial dysfunction or metabolic abnormalities, their therapeutic may rely more strongly on metabolic reprogramming and mitochondrial homeostasis [100]. Despite these encouraging findings, most evidence is derived from young rodent models that incompletely recapitulate the complexity, chronicity, and heterogeneity of human disease. Future preclinical studies should therefore incorporate aged and comorbid animal models, clinically relevant disease settings, and standardized functional evaluation to improve the translational value of experimental findings.

## 5 Clinical Translation: Evidence From Human Trials

The clinical development of MSCs‐based therapies has progressed from early exploratory studies to systematic evaluation in registered trials. Over 1500 clinical studies (ClinicalTrials.gov) have investigated MSCs across a broad range of indications, including bone and muscle, cardiovascular, immune‐mediated, neurological, respiratory, endocrine and metabolic, urological, sensory disorders, and cancers (Table 2). Several indications have advanced to Phase III trials, and a limited number of products have received regulatory approval, pointing to early signs of clinical translation.

**TABLE 2:** Clinical trials of MSCs therapy for various diseases.

Sources of MSCs	Research subjects	Clinical trial IDs	Recruitment status	Stage	Objective	Preliminary results	References
UC‐MSCs‐derived EVs	KOA	NCT06431152	Completed	Phase I	To evaluate the safety of intra‐articular injection of UC‐MSCs‐EVs for mild‐to‐moderate KOA.	No adverse events	[103]
AD‐MSCs	KOA	NCT03990805	Completed	Phase III	To evaluate the efficacy and safety of autologous AD‐MSCs in patients with severe KOA.	Alleviate joint pain and improved function	[256]
BM‐MSCs	Achilles tendinopathy	NCT02064062	Completed	Phase IIA	To evaluate the safety of autologous BM‐MSCs implantation into human diseased Achilles tendons.	Favorable safety profile	[257]
UC‐MSCs	ICM	NCT06145035	Recruiting	Phase IIA	To assess the safety, feasibility, and efficacy of UC‐MSCs.	Not yet completed	——
WJ‐MSCs	AMI	IRCT20201116049408N1	Completed	Phase II–III	To compare single versus double WJ‐MSCs transplantation for LVEF in AMI patients.	LVEF was significantly improved	[258]
WJ‐MSCs	HF	NCT05043610	Completed	Phase III	To evaluate the effects of intracoronary infusion of WJ‐MSCs on the development of HF following MI.	Reduce the incidence of heart failure and related hospitalization rates	[259]
AD‐MSCs	IHF	NCT03092284	Completed	Phase II	To assess the safety and efficacy of allogeneic AD‐MSCs injection in patients with IHF.	Safe but clinically ineffective	[260]
BM‐MSCs	AD	NCT05233774	Completed	Phase IIA	To assess the safety and preliminary efficacy of allogeneic BM‐MSCs in AD.	Safe and effective	[261]
BM‐MSCs	PD	NCT04506073	Completed	Phase II	To evaluate the efficacy of allogeneic BM‐MSCs therapy in improving motor symptoms in PD patients.	Favorable safety and tolerability	[262]
AD‐MSCs	SCI	NCT03308565	Completed	Phase I	To evaluate the safety and tolerability of autologous AD‐MSCs in SCI.	Favorable safety and tolerability	[263]
UC‐MSCs	SLE	NCT03171194	Completed	Phase I	To evaluate the safety of allogeneic UC‐MSCs therapy in SLE patients.	Safe and potentially effective	[264]
AD‐MSCs	RA	NCT03691909	Completed	Phase I/IIA	To evaluate the safety and efficacy of intravenous infusion of autologous AD‐MSCs in RA patients.	Safe and effective	[265]
UC‐MSCs	IIM	NCT04976140	Completed	Phase I/IIA	To evaluate the safety, tolerability, and efficacy of PN‐101 in patients with IIM.	Favorable safety and potential clinical efficacy	[266]
BM‐MSCs	PF	NCT02594839	Completed	Phase I/II	To evaluate the safety and efficacy of high‐cumulative‐dose allogeneic BM‐MSCs in patients with rapidly progressive PF.	Safe and well‐tolerated, with significant improvements in pulmonary function	[267]
UC‐MSCs	T2DM	NCT02302599	Completed	Phase II	To investigate the efficacy and safety of intravenous UC‐MSCs infusion in T2DM patients.	With favorable safety and high efficacy (reduce the demand for exogenous insulin and alleviate insulin resistance)	[268]
BM‐MSCs	T1DM	NCT04078308	Completed	Phase I/II	To evaluate the safety and efficacy of BM‐MSCs transplantation in patients with T1DM.	Safe and effective	[269]
BM‐MSCs	DKD	NCT02585622	Completed	Phase IB/IIA	To evaluate the safety and tolerability of BM‐MSCs therapy in subjects with DKD.	Favorable safety and tolerability	[270]
UC‐MSCs	DED	NCT05784519	Completed	Early Phase I	To evaluate the safety and efficacy of UC‐MSCs eye drops in patients with DED.	Favorable safety and tolerability	[271]
UC‐MSCs	AIS	NCT04097652	Completed	Phase I	To evaluate the safety and tolerability of intravenous UMC119‐06 in patients with AIS.	Favorable safety and tolerability	[272]
BM‐MSCs‐derived EVs	COVID‐19‐associated ARDS	NCT04493242	Completed	Phase II	To evaluate the safety and efficacy of intravenous BM‐MSCs‐derived exosomes in COVID‐19‐associated ARDS.	Favorable safety profile and reduced mortality	[273]

Abbreviations: AIS, acute ischemic stroke; DED, dry eye disease.

### 5.1 Completed and Ongoing Clinical Trials

Bone and muscle diseases are among the most extensively studied indications for MSCs therapy. Multiple Phase I–III clinical trials have demonstrated that intra‐articular administration of autologous or allogeneic MSCs alleviates pain, improves joint function, and preserves or restores cartilage in patients with KOA [256, 274, 275, 276]. While some trials report cartilage regeneration with increased cartilage thickness, others indicate prevention of cartilage deterioration rather than volume increase [256, 274, 275]. Stempeucel, an allogeneic BM‐MSCs product, has completed a Phase III trial with therapeutic benefits maintained for at least 24 months [275, 276]. In addition to KOA, autologous BM‐MSCs have demonstrated favorable safety in Achilles tendinopathy treatment, while lyophilized MSCs‐derived apoptotic vesicles have shown promise for hemostasis and alveolar bone regeneration following impacted third molar extraction [257, 277].

Cardiovascular studies have primarily focused on AMI and HF. Intracoronary transplantation of WJ‐MSCs with a booster dose improved left ventricular function in a Phase II trial, and a subsequent Phase III study reported reduced HF incidence and HF‐related hospitalizations [258, 259]. However, clinical outcomes vary according to disease subtype. AD‐MSCs failed to achieve the primary endpoint in chronic ischemic HF, whereas favorable safety and preliminary efficacy were observed in chronic nonischemic HF, suggesting that disease etiology may influence therapeutic efficacy [260, 278].

Clinical studies in neurological disorders have also provided encouraging evidence for the therapeutic potential of MSCs‐based therapies, although confirmation in larger, well‐controlled trials is still required. A randomized, double‐blind, placebo‐controlled Phase IIA trial showed that laromestrocel, an allogeneic BM‐MSCs product, slowed whole brain atrophy by 48.4% and improved cognitive function in patients with mild AD [261]. In PD, clinical benefit was observed only after repeated administration of allogeneic BM‐MSCs at the highest tested dose (10 × 10^6^ cells/kg), whereas autologous AD‐MSCs showed a favorable safety profile and tolerability profile in traumatic SCI [262]. In traumatic SCI, autologous AD‐MSCs demonstrated a favorable safety profile and good tolerability [263].

Immune‐mediated diseases have shown the strongest clinical evidence supporting MSCs‐based therapies. Allogeneic UC‐MSCs have demonstrated favorable safety and preliminary efficacy in SLE, while autologous AD‐MSCs showed favorable safety and improved joint function in RA [264, 265, 279]. Clinical studies have demonstrated the therapeutic efficacy of MSCs in graft‐versus‐host disease (GVHD) [280]. In a prospective, single‐arm Phase III trial, the MSCs‐based product Ryoncil achieved an overall response rate of 70% and a 6‐month survival rate of 69% in 54 pediatric patients (1 month–17 years) with Grade II–IV steroid‐refractory acute GVHD (SR‐aGVHD), providing the basis for subsequent regulatory approval [281]. Long‐term follow‐up further demonstrated durable efficacy through Day 180 with no new safety concerns [281]. In addition to these indications, preliminary clinical studies have also exhibited favorable safety and potential therapeutic benefits of MSCs therapy in patients with psoriasis and idiopathic inflammatory myopathy (IIM) [266, 282]. In respiratory diseases, MSCs and their derived EVs have demonstrated favorable safety and preliminary efficacy in early‐phase clinical studies, particularly in ARDS and PF, although confirmation in larger randomized trials is still required [267, 283, 284].

Early‐phase clinical studies have shown that MSCs‐based therapies are well tolerated in patients with T1DM, T2DM, DFUs, and DKD, with no major treatment‐related adverse events reported [268, 269, 270, 285]. Improvements in glycemic control have been observed in both T1DM and T2DM, whereas restoration of β‐cell function appears to be more evident in newly diagnosed T1DM than in established T2DM [268, 269]. MSCs therapy also improved wound healing in patients with DFUs, whereas evidence of efficacy in DKD remains limited [270, 285]. However, current evidence is still derived from relatively small, early‐phase studies with short follow‐up, and whether these therapeutic benefits can be sustained and reproduced in larger patient populations remains to be determined. Meanwhile, preliminary evidence also supports their use in urological and ophthalmological conditions, though these indications remain less extensively studied [271, 286, 287].

For cancer treatment, current clinical investigations have mainly focused on supportive care and prevention of disease progression. For example, UC‐MSCs administration improved liver function while maintaining a favorable safety profile in patients with HBV‐associated decompensated liver cirrhosis (DLC), findings that were further supported by a Phase I dose‐escalation trial and a real‐world cohort study using autologous BM‐MSCs [288, 289, 290].

Overall, completed clinical trials indicate that the clinical efficacy of MSCs varies considerably across disease settings [256, 257, 258, 259, 260, 261, 262, 263, 264, 265, 268, 269, 270, 271, 274, 275, 276, 277, 278, 279, 280, 281, 283, 284, 285, 286, 287]. The strongest clinical evidence currently supports their use in musculoskeletal and immune‐mediated disorders, with multiple trials demonstrating improvements in symptoms and functional outcomes [256, 257, 264, 265, 274, 275, 276, 277, 279, 280, 281]. By contrast, studies of cardiovascular, neurological, respiratory, and endocrine disorders have generally demonstrated acceptable safety and preliminary efficacy, although therapeutic responses remain variable across disease subtypes and patient populations [258, 259, 260, 261, 262, 263, 268, 269, 270, 278, 283, 284, 285]. Recent clinical studies have shifted from establishing feasibility toward optimizing treatment strategies. Ongoing trials increasingly emphasize cell‐source selection, manufacturing consistency, dosing regimens, delivery routes, and patient stratification [291]. Nevertheless, substantial heterogeneity in MSCs products, trial design, administration protocols, and clinical endpoints continues to limit cross‐study comparisons and the generation of high‐quality clinical evidence [13].

### 5.2 Safety Profile and Meta‐Analyses

Evidence from randomized controlled trials (RCTs) and meta‐analyses indicates that MSCs therapy is generally well tolerated, with no major safety concerns identified to date. A meta‐analysis of 62 RCTs involving 3546 patients found that MSCs administration was not associated with an increased risk of serious adverse events, with only transient fever, administration‐site reactions, insomnia, constipation, and fatigue observed [292]. Consistent with these findings, an updated systematic review of 42 RCTs involving 2280 patients confirmed that UC‐MSCs therapy did not increase the incidence of prespecified adverse events, including infection, malignancy, thromboembolic events, or mortality, except for a higher incidence of transient fever [293].

However, efficacy outcomes have been less consistent across different disease indications. In ICM, a recent meta‐analysis of 45 clinical trials involving 1408 patients showed that MSCs therapy improved left ventricular ejection fraction (LVEF); however, treatment effects varied across studies, and the overall certainty of the evidence was limited by methodological heterogeneity and study quality [294]. Similarly, in SR‐aGVHD, an updated meta‐analysis of four RCTs involving 650 patients showed that MSCs therapy improved the short‐term overall response rate without increasing treatment‐related adverse events, although no significant improvement in overall survival was observed [295].

Although the favorable short‐term safety profile, evidence regarding long‐term safety remains limited. Most clinical studies have follow‐up periods shorter than 2 years, and potential risks, including tumorigenicity, immunogenicity, and ectopic tissue formation, have not been adequately evaluated [296, 297].

### 5.3 Regulatory Approvals and Marketed Products

The regulatory approval of MSCs‐based products marks an important milestone in the clinical translation of MSCs therapy [298]. Between late 2024 and early 2025, the United States and China approved Ryoncil (remestemcel‐L‐rknd) and Ai Mi Mai Tuo Sai Injection (Rui Bo Sheng), respectively, as their first marketed MSCs‐based therapies for SR‐aGVHD [299, 300]. To date, more than 10 MSCs‐based products have received marketing authorization worldwide, with approvals concentrated mainly in South Korea, Japan, Canada, China, and the United States [301]. Representative products include Queencell, Cellgram AMI, Cupistem, Cartistem, NeuroNata‐R, Temcell HS, Ryoncil, and Rui Bo Sheng. Among the approved indications, SR‐aGVHD represents the most extensively validated application, with four MSCs products currently authorized for this indication [299, 301].

## 6 Challenges in Manufacturing and Standardization

Although MSCs‐based therapies have demonstrated therapeutic potential across a wide range of diseases, their clinical translation continues to face significant challenges related to cell heterogeneity, lack of manufacturing standardization, and poorly defined pharmacokinetics, which collectively constitute the core bottlenecks to widespread clinical implementation.

Source‐dependent heterogeneity is one of the primary factors affecting the consistency of MSCs products [28]. MSCs isolated from different tissues, including bone marrow, adipose tissue, and umbilical cord, exhibit inherent differences in proliferation, secretome composition, immunomodulatory capacity, and gene expression patterns [28]. Differences in culture protocols, including serum source, oxygen concentration, and passage number, further increase interbatch variability and may contribute to inconsistent clinical outcomes [302]. In addition, although in vitro expansion enables the generation of sufficient cell numbers for clinical applications, repeated passaging inevitably induces replicative senescence and the senescence‐associated secretory phenotype (SASP), leading to impaired proliferative capacity and potentially persistent proinflammatory effects after transplantation [303].

Another major challenge is the limited pharmacokinetic performance of MSCs, particularly their homing efficiency and in vivo persistence [304]. Following intravenous administration, approximately 70%–90% of MSCs become trapped within the pulmonary capillary network because of mechanical sequestration, whereas typically fewer than 1%–5% of infused cells reach the target tissue [304, 305]. Moreover, MSCs generally exhibit limited persistence in vivo, with most cells being cleared by the immune system within days to weeks after administration [306]. Current evidence indicates that their therapeutic effects are mediated predominantly through transient paracrine mechanisms rather than long‐term engraftment. Consequently, repeated administration is often required to compensate for limited persistence, which may increase treatment costs and the potential risk of immunogenicity [307]. To date, the optimal route of administration, cell dose, and treatment schedule have not been standardized.

Safety remains an important factor that needs to be taken into consideration in MSC‐based therapies. Although MSCs are generally regarded as having low immunogenicity and good tolerability, accumulating evidence suggests that potential risks warrant continued attention [308]. Besides the pulmonary embolism associated with intravenous cell entrapment, their potential protumorigenic effects within the TME have also raised concern. MSCs may facilitate tumor cell proliferation, drug resistance, and metastasis through mitochondrial transfer, secretion of bioactive mediators, or differentiation into cancer‐associated fibroblasts [89]. Furthermore, although some studies have attempted to generate immortalized MSCs via lentiviral transfection of human telomerase reverse transcriptase (hTERT) or to optimize culture conditions by adding GFs, these strategies introduce new problems, including increased tumorigenic risk, higher costs, and greater complexity of ethical regulation [309, 310].

Regulatory harmonization represents another major obstacle to clinical translation. Although the United States, the European Union, Japan, and China have each established regulatory frameworks for MSCs‐based therapies, substantial differences remain in product characterization, quality control, and potency assessment [310, 311]. Requirements for cell activity, purity, potency, proliferative capacity, genomic stability, and microbiological testing vary across regulatory agencies, thereby complicating cross‐study comparisons and international product registration [312]. For example, despite encouraging progress in MSCs therapy for KOA, large‐scale manufacturing and the transition from laboratory research to industrial production remain major challenges [313]. Furthermore, with the exception of Mesoblast's Ryoncil, no other commercial MSCs product has received regulatory approval in the United States [298].

Overall, MSCs should not be considered inherently safe or innocuous. They are active biological products that require rigorous risk‐benefit assessment. Addressing the translational bottlenecks described above will be essential for developing clinical‐grade MSCs products that meet regulatory and therapeutic requirements.

## 7 Strategies to Enhance MSCs Potency

**FIGURE 3:**
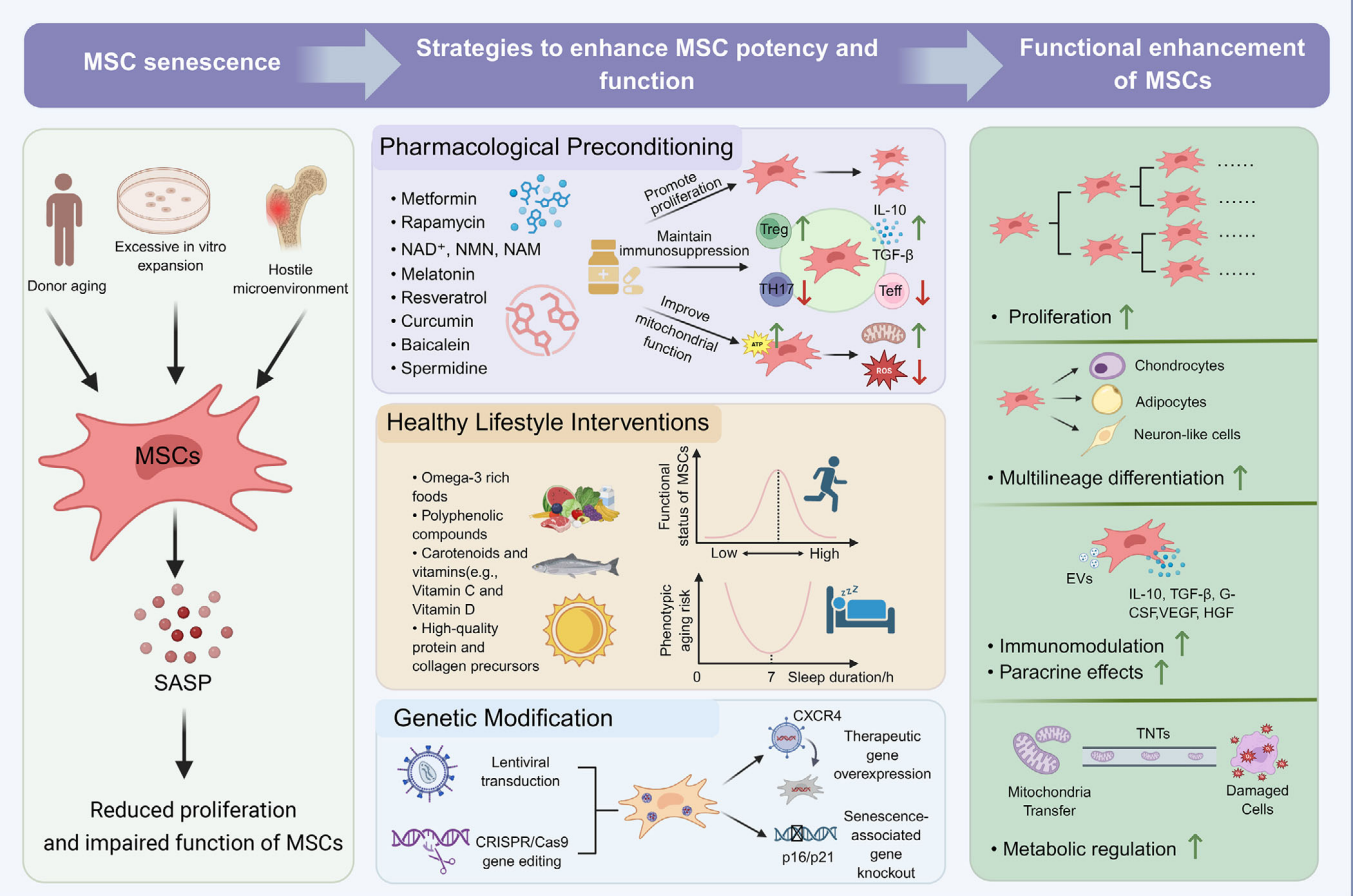
Strategies to enhance MSCs potency and function. Donor aging, excessive in vitro expansion, and the diseased microenvironment contribute to MSCs senescence, leading to reduced proliferative capacity and impaired biological functions. Pharmacological preconditioning, healthy lifestyle interventions, and genetic modification can enhance MSCs proliferation and improve their multilineage differentiation, paracrine activity, immunomodulatory capacity, and metabolic regulation, thereby enhancing their therapeutic potential.

Despite their broad therapeutic potential and diverse biological functions, the clinical efficacy of MSCs is often limited by progressive functional decline during manufacturing and after transplantation. Donor ageing, extensive in vitro expansion, and the hostile microenvironment of injured tissues can impair MSCs function by inducing cellular senescence and altering their secretory profile [11, 314]. To overcome these limitations, a variety of strategies have been developed to improve MSCs potency, enhance product consistency, and expand their therapeutic applications. These strategies mainly include preconditioning, genetic engineering, and cell‐free therapies based on MSCs‐derived EVs [314, 315, 316] (Figure 3).

### 7.1 Preconditioning and Priming

Preconditioning is one of the most widely investigated approaches for enhancing the therapeutic efficacy of MSCs [316]. Exposure to hypoxic, inflammatory, or pharmacological stimuli can induce adaptive responses that enhance MSCs survival, migratory capacity, immunomodulatory activity, and secretion of therapeutic factors [317, 318]. For example, hypoxic preconditioning improves stemness and angiogenic potential, whereas inflammatory priming with IFN‐γ and TNF‐α strengthens the immunomodulatory properties of MSCs [316]. Pharmacological preconditioning has also shown considerable promise. Agents such as rapamycin, metformin, spermidine (SPD), NAD^+^ supplements, and resveratrol have been reported to improve mitochondrial function, resistance to oxidative stress, proliferative capacity, and regenerative potential, thereby enhancing the therapeutic efficacy of MSCs in preclinical disease models [319, 320, 321, 322, 323, 324, 325, 326, 327, 328, 329, 330, 331, 332, 333, 334, 335] (Table 3).

**TABLE 3:** Mechanisms and roles of pharmacological interventions that regulate MSCs quantity and function.

Pharmacological agent	Functional regulation of MSCs	Target/pathway	Disease applications	References
Metformin	Promote adipogenesis and immunosuppression of human UC‐MSCs.	PPARγ↑, FABP4↓; IL‐6↓, MCP‐1↓, COX‐2↓	**——**	[320]
	Enhance osteogenic differentiation of PL‐MSCs.	Activate the AMPK signaling pathway.	OP	[321]
	Promote the osteogenic differentiation of BM‐MSCs.	Regulate miR‐181a‐5p/PAI‐1 axis; Modulate macrophage M2 polarization.	Age‐related bone regeneration diseases (e.g., OP, fractures)	[322, 323]
	Promote differentiation of GMSCs into neuron‐like cells.	ATP5F1↑, ATP5J↑, NDUFS3↑, GLUD1↑	Central nervous system disorders (e.g., SCI, PD, AD)	[324]
	Promote the proliferation and osteogenic differentiation of BM‐MSCs.	Regulate ROS–AKT–mTOR axis.	DM	[326]
Metformin and vitamin D	Regulate the inflammatory response and autophagy process of AD‐MSCs.	IL‐6↓, TNF‐α↓; HSP60↓, HSP70↑, ATG12↑, LC3B I↑, LC3B II↑	Obesity‐related metabolic disorders.	[325]
Rapamycin	Promote the immune regulation and tissue repair capability of PL‐MSCs.	Autophagy↑, TGF‐β↑, IL‐6↓, IL‐8↑	Bone healing	[327]
NMN	Rejuvenate mitochondrial function and attenuate BM‐MSCs senescence.	Activate NAD+/Sirt3 signaling pathway.	——	[328]
NAD+	Reprogram the immunomodulatory function of BM‐MSCs.	NAMPT↑, NAD+↑, HIF‐1α↑	Inflammatory diseases	[329]
NMN	Promote BM‐MSCs self‐renewal with strengthened osteogenesis and reduce adipogenesis.	Upregulate SIRT1.	OP	[331]
NAM	Restore the mitochondrial function of senescent BM‐MSCs.	Reactivate NAD+/Sirt1 axis.	——	[330]
SPD	Maintain proliferation and differentiation of human UC‐MSCs.	SIRT3↑, ROS↓	——	[332]
Resveratrol	Promote proliferation, osteogenic differentiation, and immunomodulation of GMSCs.	Reduce the expression of TLR4, TNFα, and NF‐κB and activate ERK/Wnt pathway.	Periodontal diseases	[333]
Curcumin	Alleviate senescence of BM‐MSCs and improve mitochondrial dysfunction.	Promote mitophagy.	Aging‐related diseases	[334]
Melatonin	Promote the proliferation, migration, and differentiation of human UC‐MSCs.	Activate the PI3K/AKT pathway.	T2DM	[335]

Lifestyle‐related interventions have also emerged as potential modulators of MSCs function. Accumulating evidence suggests that regular physical activity, healthy dietary patterns, and adequate sleep help preserve MSCs viability and functional capacity, highlighting the importance of the systemic host environment for MSCs‐based therapy [336]. Mechanistically, these interventions have been associated with improved metabolic homeostasis, reduced oxidative stress, enhanced mitochondrial function, and preservation of regenerative capacity [337, 338, 339, 340, 341, 342, 343, 344, 345, 346, 347]. For example, exercise can enhance MSCs proliferation and regenerative potential, whereas dietary factors such as vitamin D supplementation may support osteogenic differentiation and reduce oxidative stress [337, 338, 340, 341]. Similarly, maintenance of a normal circadian rhythm preserves the proliferative and differentiation capacity of MSCs, whereas circadian disruption has been associated with impaired tissue repair and accelerated progression of cardiovascular and metabolic disorders [346, 347]. Although most evidence remains preclinical, lifestyle optimization may represent a complementary strategy for maintaining MSCs functionality and improving therapeutic outcomes.

### 7.2 Genetic Engineering

Genetic modification has emerged as an effective strategy for augmenting MSCs functionality. Using lentiviral transduction or CRISPR/Cas9‐mediated gene editing, engineered MSCs can enable sustained overexpression of therapeutic transgenes or targeted disruption of senescence‐associated genes, thereby enhancing regenerative, immunomodulatory, and tissue‐protective functions [348]. Several recent studies have evaluated this approach in diverse disease models. CXCR4 overexpression enhanced human UC‐MSCs homing to injured brain tissue in a rat model of cardiac arrest and improved neurological recovery through exosome‐mediated inhibition of neuronal pyroptosis [349]. In a gastric cancer (GC) xenograft model, MSCs engineered to coexpress CXCR4 and LIGHT (TNFSF14) showed enhanced tumor tropism and antitumor activity [350]. In addition, glycogen engineering, which modifies the glycogen‐synthesis pathway, improved MSCs survival under nutrient deprivation and enhanced therapeutic efficacy in PF [351]. Before clinical application, however, transgenic MSCs require rigorous evaluation of off‐target effects, genomic stability, and tumorigenic risk.

### 7.3 MSCs‐Derived EVs as Alternatives

Accumulating evidence indicates that MSCs‐derived EVs can retain most of the biological activities of their parent cells and may therefore provide a cell‐free alternative to MSCs therapy [77, 352]. Compared with live‐cell products, EVs have lower immunogenicity, cannot proliferate, and may penetrate tissues more readily. They may also offer practical advantages in storage, transportation, quality control, standardization, and large‐scale production [353, 354]. Optimization strategies developed for parental MSCs may also be applicable to their EVs. Preconditioning or genetic modification can alter EV cargo and enrich therapeutically active miRNAs, proteins, and lipids, thereby enhancing biological activity [355]. However, clinical translation remains constrained by challenges in large‐scale good manufacturing practice production, product consistency, potency assessment, biodistribution, and pharmacokinetics [356]. With the continuous improvement of related technologies and quality control systems, EVs derived from MSCs are expected to become an important supplement to MSCs therapy and further promote the development of cell‐free regenerative medicine.

## 8 Conclusions and Future Perspectives

Over recent decades, MSCs research has advanced from basic characterization of multilineage differentiation to the development of clinically manufactured cell products [27, 30]. Their therapeutic effects are now attributed mainly to modulation of inflammatory and regenerative microenvironments, rather than durable engraftment or direct tissue replacement [357, 358]. This shift has driven the development of MSCs‐based therapeutics, particularly engineered MSCs and MSCs‐derived EVs [77, 349, 352]. The approval of Ryoncil for pediatric SR‐aGVHD represents an important clinical and regulatory milestone [299]. It demonstrates that an MSCs product can achieve successful clinical translation under established manufacturing, quality control, and regulatory standards. However, this approval is specific to a particular MSCs product and clinical indication, and should not be generalized to other MSCs products or clinical indications.

A major challenge for clinical translation is to establish reliable approaches for assessing MSCs product potency in the intended clinical setting. MSCs function is influenced by multiple factors, including tissue source, donor characteristics, manufacturing processes, culture conditions, passage number, and cryopreservation, resulting in substantial product‐to‐product variability [28, 302, 304]. Conventional identity criteria alone are insufficient to predict therapeutic performance, and products with comparable surface phenotypes may therefore differ in immunomodulatory, angiogenic, reparative, or metabolic activity [30, 312, 359]. Accordingly, potency assays should reflect the intended mechanism of action and correlate with clinically relevant outcomes. Because the functional requirements of MSCs vary across disease indications, potency assessment should be tailored to the intended indication rather than relying on a single testing strategy. The emerging framework of critical quality attributes (CQAs) represents an important step toward standardized MSCs product characterization [312]. However, its ability to predict product potency and clinical performance remains to be validated across manufacturing batches and clinical cohorts. Continued efforts to standardize manufacturing, product characterization, and potency assessment will improve product consistency and enhance the comparability of clinical studies.

Several key aspects of MSCs behavior after administration remain poorly understood. These include the persistence of transplanted cells in vivo and the relative contributions of viable cells, apoptotic cells, soluble mediators, MSCs‐derived EVs, and host responses to therapeutic efficacy [353, 360]. Treatment response is also likely to depend on disease stage, patient characteristics, concomitant therapies, dose, and treatment schedule. Addressing these questions will require adequately powered, well‐designed clinical trials with harmonized clinical endpoints and trial designs informed by the proposed mechanism of action. Integration of pharmacodynamic biomarkers, longitudinal immune and metabolic profiling, and product‐tracking strategies will provide mechanistic insight into treatment responses and support biomarker‐guided patient stratification. Long‐term follow‐up will be important to determine the durability of clinical benefit and to monitor delayed immunogenicity, ectopic tissue formation, and potential tumorigenicity [361]. Comprehensive reporting of manufacturing processes and product characteristics will also help determine whether variable clinical outcomes reflect product‐related differences or limitations in patient selection, dosing strategies, or trial design.

Future development should move toward disease‐specific MSCs products tailored to the biological characteristics and therapeutic requirements of individual indications. Selection of cell source, donor characteristics, culture conditions, priming strategies, genetic modification, and delivery routes should be guided by disease biology and the intended therapeutic mechanism. Single‐cell and spatial analyses may help define functional heterogeneity and characterize manufacturing‐associated changes in MSCs composition. Their clinical utility, however, will depend on robust functional validation and correlation with clinically relevant outcomes. MSCs‐derived EVs offer advantages in formulation, storage, and tissue delivery. However, cargo heterogeneity, dose definition, biodistribution, and potency assessment remain major challenges. Addressing these issues will be essential for their clinical translation. Ultimately, successful clinical translation will depend on integrating mechanistic research with standardized manufacturing, clinically relevant potency assessment, rational patient selection, and appropriate regulatory frameworks.

## Author Contributions

**Dongmei Xue and Lei Wu** wrote this manuscript and created figures and tables. **Dongmei Xue and Lei Wu** performed the literature search. **Yongsheng Li and Lei Wu** revised the manuscript. **Yongsheng Li and Lei Wu** provided guidance throughout the preparation of the manuscript. All the authors read and approved the final manuscript.

## Funding

This work was supported by National Natural Science Foundation of China (Nos. 82572105, 82271885, and 32470971), Major Project of Joint Medical Research of Science and Health in Chongqing (2025DBXM006), Middle‐Aged and Young Medical Talent Project of Chongqing, National Outstanding Youth Reserve Talent Training Project (No. HBRC202406), Natural Science Foundation of Chongqing (No. 2024NSCQ‐KJFZMSX0059), Chongqing Natural Science Foundation Innovation and Development Joint Fund Project (No. CSTB2025NSCQ‐LZX0004), and Funding for Chongqing Young and Middle‐Aged Medical Excellence Team.

## Ethics Statement

The authors have nothing to report.

## Conflicts of Interest

The authors declare no conflicts of interest.

## Data Availability

The authors have nothing to report.
